# Aetiology of hospitalized fever and risk of death at Arua and Mubende tertiary care hospitals in Uganda from August 2019 to August 2020

**DOI:** 10.1186/s12879-022-07877-3

**Published:** 2022-11-21

**Authors:** Paul W. Blair, Kenneth Kobba, Francis Kakooza, Matthew L. Robinson, Emmanuel Candia, Jonathan Mayito, Edgar C. Ndawula, Abraham J. Kandathil, Alphonsus Matovu, Gilbert Aniku, Yukari C. Manabe, Mohammed Lamorde

**Affiliations:** 1 John Hopkins University School of Medicine Division of Infectious Diseases, Baltimore, MD USA; 2 Henry M. Jackson Foundation for the Advancement of Military Medicine, Inc., 6720A Rockledge Dr., Bethesda, MD USA; 3 Infectious Diseases Institute, Makerere University, Kampala, Uganda; 4 Mubende Regional Referral Hospital, Mubende, Uganda; 5 Arua Regional Referral Hospital, Arua, Uganda

**Keywords:** Acute febrile illness, Uganda, Epidemiology, Mubende, Emerging communicable diseases

## Abstract

**Background:**

Epidemiology of febrile illness in Uganda is shifting due to increased HIV treatment access, emerging viruses, and increased surveillance. We investigated the aetiology and outcomes of acute febrile illness in adults presenting to hospital using a standardized testing algorithm of available assays in at Arua and Mubende tertiary care hospitals in Uganda.

**Methods:**

We recruited adults with a ≥ 38.0 °C temperature or history of fever within 48 h of presentation from August 2019 to August 2020. Medical history, demographics, and vital signs were recorded. Testing performed included a complete blood count, renal and liver function, malaria smears, blood culture, and human immunodeficiency virus (HIV). When HIV positive, testing included cryptococcal antigen, CD4 count, and urine lateral flow lipoarabinomannan assay for tuberculosis. Participants were followed during hospitalization and at a 1-month visit. A Cox proportional hazard regression was performed to evaluate for baseline clinical features and risk of death.

**Results:**

Of 132 participants, the median age was 33.5 years (IQR 24 to 46) and 58.3% (n = 77) were female. Overall, 73 (55.3%) of 132 had a positive microbiologic result. Among those living with HIV, 31 (68.9%) of 45 had at least one positive assay; 16 (35.6%) had malaria, 14 (31.1%) tuberculosis, and 4 (8.9%) cryptococcal antigenemia. The majority (65.9%) were HIV-negative; 42 (48.3%) of 87 had at least one diagnostic assay positive; 24 (27.6%) had positive malaria smears and 1 was Xpert MTB/RIF Ultra positive. Overall, 16 (12.1%) of 132 died; 9 (56.3%) of 16 were HIV-negative, 6 died after discharge. High respiratory rate (≥ 22 breaths per minute) (hazard ratio [HR] 8.05; 95% CI 1.81 to 35.69) and low (i.e., < 92%) oxygen saturation (HR 4.33; 95% CI 1.38 to 13.61) were identified to be associated with increased risk of death.

**Conclusion:**

In those with hospitalized fever, malaria and tuberculosis were common causes of febrile illness, but most deaths were non-malarial, and most HIV-negative participants did not have a positive diagnostic result. Those with respiratory failure had a high risk of death.

**Supplementary Information:**

The online version contains supplementary material available at 10.1186/s12879-022-07877-3.

## Introduction

Systemic causes of fever are notoriously challenging to differentiate clinically in tropical regions including sub-Saharan Africa (SSA), ranging from malaria to Ebola virus disease [1]. Clinicians often rely on local epidemiology to guide empiric treatment decisions. However, during the past decade, the epidemiology of hospitalized, acute febrile illness (AFI) may have shifted in Uganda due to improved access to HIV treatment [2] and increased detection of zoonotic infections [3]. Additionally, disease recognition is largely limited to what well performing diagnostics are available.

A few decades ago during the early U.S. President’s Emergency Plan for AIDS Relief (PEPFAR) era in SSA, HIV coinfections were responsible for the majority of hospitalizations with severe febrile illness and associated with high mortality [2, 4–6]. The introduction of antiretroviral therapy (ART) and the World Health Organization (WHO) recommendation to ‘treat all’ regardless of CD4 T-cell count has reduced mortality [7–9]. However, late presentation remains a tenacious problem with significant opportunistic infection related early mortality after the initiation of ART [10]. Currently, Uganda national guidelines recommend universal HIV testing and ART ideally before the development of AIDS. Treating pre-emptively with ART [11], screening for cryptococcal antigen positivity, and urine lipoarabinomannan (LAM) testing in all hospitalized adults with HIV have been shown to decrease early mortality [12]. Since this shift to improved testing and treatment, the effect on the epidemiology of acute febrile illness among hospitalized adults in Uganda has yet to be fully characterized.

Due to high biodiversity and population density, many emerging and re-emerging zoonotic infections have been identified in Uganda particularly near the Rift Valley, further expanding the differential diagnosis of undifferentiated fever. With increasing surveillance, more cases of viral hemorrhagic fevers (VHF), including autochthonous and imported Ebola virus disease outbreaks, Crimean–Congo hemorrhagic fever (CCHF) and Rift Valley fever (RVF), have been detected in Uganda [3, 13–16]. Therefore, our acute febrile illness cohort was developed to characterize most common causes of acute febrile illness in Uganda and to determine the frequency of febrile illness due to unknown etiologies. An Ebola virus disease outbreak at Mubende Regional Referral Hospital (RRH) due to Sudan virus was declared on 20th of September 2022. Given the difficulty of distinguishing Ebola virus disease from other causes of acute febrile illness, we describe here the clinical epidemiology of results to date of the acute febrile illness cohort at Mubende RRH that preceded this outbreak. We describe herein the characteristics of hospitalized patients with AFI at two tertiary care centers in Uganda and the diagnostic yield of available testing.

## Methods

### Study design

From August 2019 to August 2020, we recruited adults 18 years of age or older presenting to Mubende and Arua Regional Referral Hospitals (RRHs) emergency or outpatient departments designated to be hospitalized with a measured or reported temperature ≥ 38.0 °C occurring within the past 48 h or a clinical history consistent with fever within 48 h of presentation. There were 255 participants consecutively screened with 97 enrolled at Mubende RRH and 35 enrolled at Arua RRH (Additional file 1: Figure S1). Participants with COVID-19 diagnoses were in separate designated areas, required scarce personal protective equipment, and were not enrolled. Exclusion criteria included being hospitalized for ≥ 72 h, receipt of antibiotics, previous participation in the study, being a prisoner, and being a psychiatric patient.

### Study setting

The two tertiary care hospitals were chosen as study sites because both sites are located in regions with different ecology and serve different populations. Mubende RRH (bed capacity 173) is located in central Uganda and serves a district population of over 688,000 people but the catchment area goes beyond the district it serves [17]. Mubende is situated on a major road between Democratic Republic of the Congo and the capital of Uganda, Kampala. Arua RRH (bed capacity 323) is located in the more arid northwest Uganda (proximal to borders with Democratic Republic of the Congo and with South Sudan), serves over 785,000 people in the districts, and receives referrals from neighbouring countries [18].

### Data collection

After informed consent, participants provided information about sociodemographic and comorbid medical conditions at enrolment. History suggestive of exposure to zoonotic infections was obtained including area of residence, occupation, environmental exposures, and healthcare exposure [19]. Also, recent antibiotic use was documented, relying on patient self-report or any clinical documents they possessed. Vital signs were recorded including temperature, heart rate, respiratory rate, blood pressure, and pulse oximetry; a Glasgow Coma Score (GCS) was calculated. Participants were prospectively followed until hospital discharge. Inpatient antibiotic usage, vital signs monitoring, intensive care unit admission, length of stay, timing of receipt of infectious disease test results, discharge diagnosis, and vital status at discharge were recorded. Upon discharge, participants returned after 3–8 weeks and were interviewed on duration of illness and antimicrobial use and serum, whole blood, and plasma stored for future assessment of seroconversion. If participants were unable to follow-up in-person, they were contacted via telephone to determine vital status.

### Clinical laboratory testing

Laboratory tests that were conducted included complete blood count, serum creatinine, serum alanine transferase (ALT), serum aspartate transferase (AST), blood cultures (one aerobic bottle, Bactec 9050, BD, NJ, United States), hepatitis A IgM (Vaxpert, Inc., Deira Dubai, UAE), hepatitis B sAg (SD Bioline, Abbott diagnostics, Gyeonggi-do, Republic of Korea), and HIV testing with consent (Determine HIV1/2: Abbott, OK, USA; Stat-Pak, Chembio Diagnostics Systems, Inc, NY, USA; SD Bioline: Gyeonggi-do, Republic of Korea), malaria rapid test (CareStart malaria Pf HRP2 antigen RDT), and thick malaria smears. A malaria smear result was stratified by any parasitaemia or ≥ 3000 parasites/μL [20, 21]. If there was clinical suspicion, testing for tuberculosis was performed using PCR sputum testing (Xpert MTB/RIF Ultra, Cepheid, Sunnyvale, CA, USA). If HIV testing was positive, serum cryptococcal antigen, urine lipoarabinomannan (LAM; Alere Waltham, MA, United States), and plasma CD4 count (BD Facs Calibur, BD, NJ, United States) were measured. HIV testing and counselling were performed per Uganda’s national guidelines.

### Data analysis

We estimated that a target sample size of 365 patients across sites would estimate the frequency of uncommon endemic viral infections with a 5% prevalence using a precision of 0.025 at 95% confidence. Here we report initial findings from an accrued sample size of 132 participants enrolled to date to provide clinical epidemiologic estimates, particularly in the context of the development of the Ebola virus disease outbreak in Mudende district, Uganda that started in September 2022. Summary statistics were performed for baseline demographics and prevalence of difference diagnoses. Baseline demographics, symptom prevalence, physiologic parameters, clinical laboratory results, quick sequential organ failure assessment (qSOFA; range, 0 [best] to 3 [worst] points) scores [22], and subsequent diagnoses made by the research physicians on-site were summarized and stratified by HIV diagnosis and the presence of a laboratory-confirmed acute infectious disease diagnosis. We stratified these descriptive statistics between those living with HIV or were newly diagnosed with HIV and those that were HIV-negative. Kaplan–Meier curve of survival stratified by HIV status. The logrank test was also performed to evaluate for a survival different with HIV. Individuals lost to follow-up after discharge were censored at hospital discharge. After checking the proportional hazards assumption, exploratory univariate Cox regression was performed to evaluate for a survival difference associated with baseline demographics, HIV status (including both new and known), physiologic parameters, and laboratory parameters. The qSOFA (quick Sepsis Related Organ Failure Assessment) score dichotomized at a score of 2 and components of the qSOFA score were also independently fit. Dichotomized parameters were used at qSOFA score cut offs (i.e., respiratory rate > 21 breaths per minute, Glasgow coma scale < 15, and systolic blood pressure 100 mmHg) [22]. Exploratory bivariate model p-values were not corrected for multiple comparisons due to a limited sample size. Adjusted regression models were performed using parameters with bivariate regression model p-values < 0.002, a Bonferroni correction cutoff. Harrell’s C-statistic was calculated to determine the performance of these unadjusted and adjusted final models [23]. The sample set was not split into derivation and validation sets due to limited sample size. Analyses were performed using Stata, version 16.0 (StataCorp LLC, College Station, TX, USA), and figures were created using Stata or R, version 4.0.1 (R Foundation). A map of districts represented was created using ArcGIS ArcGIS Online (Redlands, CA) [24].

## Results

### Participant characteristics

Among the participants (35 at Arua RRH and 97 at Mubende RRH), the median age was 33.5 years (interquartile range [IQR]: 24.0 to 46.0); 77 (58.3%) of 132 were female (Table 1). Participants presented from 11 districts in Uganda (Additional file 1: Figure S2). Participants were enrolled at a median 4 days (IQR: 3 to 7 days) after onset of symptoms which did not differ significantly by HIV status (Table 2). The majority of those lost to follow-up (3 from Arua RRH and 11 from Mubende RRH), were male (71.4%; 11 of 14 participants) and the median age was 31.5 years (IQR: 27 to 50 years). 27 (20.5%) of 132 participants had a qSOFA severity score of 2 or greater. The most common symptoms included headache (76.0%), and anorexia (58.9%), and nausea or vomiting (58.9%) (Additional file 1: Table S1). The majority (68.2%) of patients had no known prior medical history. The median white blood count was 4.8 × 10^3^ cells/uL with a median 129.0 × 10^3^ platelets/uL (Additional file 1: Table S2).

**Table 1:** Baseline demographic and physiologic vital sign characteristics

Characteristic	Total (N = 132)	Arua RRH (N = 35)	Mubende RRH (N = 97)	HIV-negative (N = 87)	HIV-positive (N = 45)
Female sex—no. (%)	77 (58.3)	18 (51.4)	59 (60.8)	50 (57.5)	27 (60.0)
Age—years, median (IQR)	33.5 (24.0, 46.0)	31.0 (24.0, 47.5)	35.0 (24.0, 45.0)	30.0 (22.0, 44.5)	38.0 (30.0, 48.0)
Past medical history—no. (%)					
COPD or asthma	1 (0.8)	0 (0.0)	1 (1.0)	1 (1.1)	0 (0.0)
Diabetes mellitus	5 (3.8)	2 (5.7)	3 (3.1)	3 (3.4)	2 (4.4)
Sickle cell disease/thalassemia	3 (2.3)	2 (5.7)	1 (1.0)	3 (3.4)	0 (0.0)
Duration of symptoms—days, median (IQR)	4.0 (3.0, 7.0)	4.0 (3.0, 7.0)	4.0 (3.0, 7.0)	4.0 (3.0, 7.0)	5.0 (3.0, 7.0)
Physiologic parameters—median (IQR)					
Heart rate (beats per minute)	109.0 (92.8, 119.3)	103.0 (90.5, 113.5)	110.0 (94.0, 122.0)	107.0 (93.0, 119.5)	111.0 (93.0, 118.0)
Temperature (°C)	38.3 (38.0, 38.8)	38.300 (38.0, 38.8)	38.2 (38.0, 38.8)	38.3 (38.0, 38.8)	38.1 (38.0, 38.7)
Systolic blood pressure (mmHg)	110.5 (98.0, 122.0)	111.0 (95.0, 119.0)	111.0 (100.0, 122.0)	110.0 (100.0, 119.0)	114.0 (98.0, 127.0)
Diastolic blood pressure (mmHg)	68.0 (60.0, 78.0)	68.0 (60.5, 74.8)	68.0 (60.0, 78.0)	68.0 (59.0, 75.0)	69.0 (63.0, 80.0)
Respiratory rate (breaths per minute)	20.0 (18.0, 24.0)	20.0 (18.0, 26.0)	20.0 (18.0, 24.0)	22.0 (18.0, 25.5)	20.0 (18.0, 24.0)
Oxygen saturation (%)	98.0 (96.0, 99.0)	98.0 (96.0, 98.5)	98.0 (96.0, 99.0)	98.0 (96.0, 98.0)	98.0 (96.0, 99.0)
Glasgow coma scale	15.0 (15.0, 15.0)	15.0 (15.0, 15.0)	15.0 (15.0, 15.0)	15.0 (15.0, 15.0)	15.0 (15.0, 15.0)
qSOFA score ≥ 2—no. (%)	27 (20.5)	8 (22.9)	19 (19.6)	19 (21.8)	8 (17.8)

RRH: Regional Referral Hospital; IQR: interquartile range; qSOFA: quick sequential organ failure assessment

**Table 2:** Diagnostic test results stratified by HIV status and outcome

Diagnostic test	Total (n = 132)	HIV status			Outcome		
		HIV-negative (n = 87)	HIV-positive (n = 45)	p-value	Survived (n = 102)	Died (n = 16)	p-value
Malaria antigen RDT—no. (%)	39 (29.5)	24 (27.6)	15 (33.3)	0.493	33 (32.4)	1 (6.7)	**0.037**
Malaria smear qualitative result—no. (%)							
> 0 parasites/HPF	40 (30.3)	24 (27.6)	16 (35.6)	0.43	27 (28.1)	0 (0.0)	0.35
≥ 3000 parasites/HPF	12 (9.1)	10 (11.5)	2 (4.4)	0.22	10 (9.8)	0 (0.0)	
Missing values	10	6	4		6	1	
Serum cryptococcal antigen—no. (%)	–	–	5 (11.1)	–	4/31 (12.9)	1/7 (14.3)	0.922
Urine lipoarabinomannan—no. (%)	–	–	14 (31.1)	–	9/31 (29.0)	4/9 (44.4)	0.157
Sputum GeneXpert—no./total samples (% of cohort)	2/14 (14.3)	1/8 (12.5)	1/6 (16.7)	0.825	2/9 (22.2)	0/3 (0.0)	0.371
Serum hepatitis A IgM—no. (%)	9 (6.9)	1 (3.3)	8 (9.2)	0.139	9 (8.8)	0 (0.0)	0.216
Missing values	1	0	1				
Serum hepatitis B sAg—no. (%)	7 (5.3)	5 (5.7)	2 (4.4)	0.752	5 (4.9)	1 (6.2)	0.819
Blood culture positivity*	9 (6.8)	4 (4.6)	5 (11.1)	0.273	5 (4.9)	2 (12.5)	0.241

RDT: rapid diagnostic test; HPF: high powered fever; sAg: surface antigen

*Excluding common contaminants

### Characteristics of HIV-infected participants

Overall, 45 (34.1%) were HIV-infected; median CD4 count was 198.0 (IQR: 72.0, 347.0) in the setting of acute illness. There were 8 (17.8%) of 45 participants with a CD4 < 50. Sixteen (35.6%) of 45 were not known to have HIV; 10 were started on ART during hospitalization or within a month after hospitalization. The remaining 7 included 3 who died, 2 who had an unknown treatment history, 1 who was resistant to starting, and 1 who eloped from the hospital and was lost to follow-up. Seventeen (60.0%) of 29 known to have HIV were on ART prior to hospital presentation. Individuals with HIV were older (median 38.0 years; IQR: 30.0, 48.0) compared to those without HIV (median 30.0; IQR: 22, 44.5) p = 0.005, otherwise, they had similar baseline demographics (Table 1, Additional file 1: Table S3).

### Microbiologic results

**Fig. 1:**
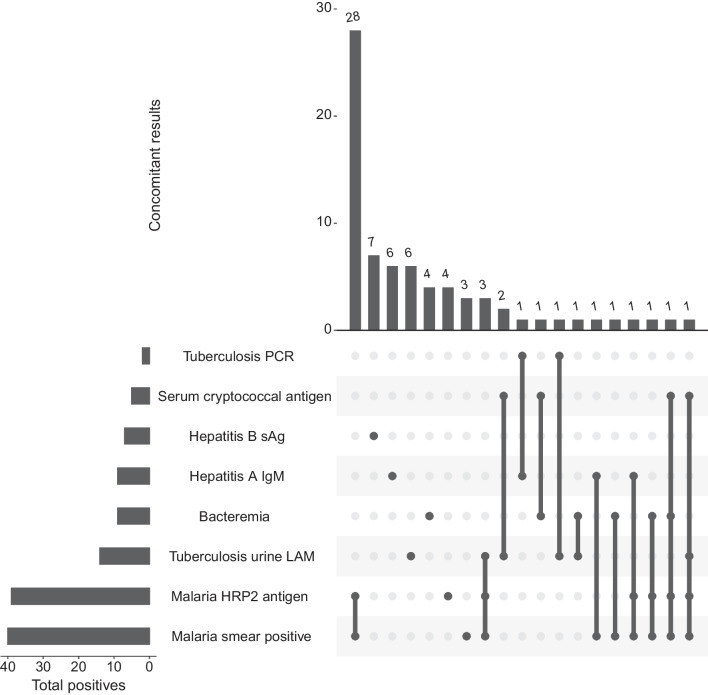
Diagnostic UpSet plot demonstrating the frequency of participants with one or more overlapping positive microbiologic results within the cohort. HRP2: *Plasmodium falciparum* antigen histidine rich protein 2; sAg: surface antigen; LAM: lipoarabinomannan

Overall, 73 (55.3%) of 132 had a positive microbiologic result. The most common positive test results were a malaria thick smear (40/132; 30.3% participants > 0 parasite/high powered field) or malaria rapid diagnostic test (39/132; 29.6% participants). Thirty-five of 40 (87.5%) positive malaria smears were also positive on the malaria RDT (Fig. 1). The second most common diagnosis was tuberculosis from a positive urine LAM (14/45; 31.1% among those with HIV, 10.6% overall cohort) or sputum GeneXpert (2/14; 14.3% among tested, 1.5% overall cohort). Bacteraemia, excluding common contaminants, was identified in 9 (6.8%) of participants. Blood cultures grew *Streptococcus pneumoniae* (n = 4), and an additional isolate each (n = 1) of *Citrobacter freundii*, *Streptococcus* spp., *Staphylococcus aureus*, *Salmonella* species, and *Proteus vulgaris*. One culture grew common contaminant coagulase negative staphylococcus, and another grew *Micrococcus luteus*.

### Microbiologic results among patients with HIV

Among those living with HIV, 31 (68.9%) of 45 had at least one positive microbiologic assay. Microbiologic and rapid diagnostic test results among those with newly diagnosed or known HIV included positive results for malaria (smear: n = 16, 35.6%; RDT: n = 15, 33.3%). There were 5 (11.1%) with cryptococcal antigenemia, 8 (9.2%) with evidence of hepatitis A and 2 (4.4%) with evidence of hepatitis B (Table 2). Bacteraemia occurred in 5 (11.1%) of 45 patients due to *Streptococcus pneumoniae* (n = 2), *Staphylococcus aureus*, *Salmonella* species, or *Proteus vulgaris* (Table 2). Fifteen (33.3%) of 45 participants with HIV also had TB; 1 out of 6 participants with sputum samples that tested positive for tuberculosis on Xpert and 14 (31.1%) had positive urine LAM results.

### Fatal cases

At 1-month follow-up, 16 (13.6%) of 118 died; 10 in hospital and 6 after discharge or hospital transfer. Death occurred a median of 3.0 days (IQR: 1 to 7 days) from enrolment. The median age among those that died was 38.0 years (IQR: 29 to 42.5, range: 18 to 80), and 7 (37.5%) had HIV (Additional file 1: Table S4). Among those who died, the most common presenting symptom was cough (n = 9, 56.2%).

At initial presentation, those that died were more tachypnoeic (median 24.5 breaths per minute, IQR: 22 to 31 breaths per minute) compared to survivors (median 20 breaths per minute; IQR: 18 to 24 breaths per minute). Fatal cases also had lower initial oxygen saturation (median 95.5%; IQR: 92.3 to 98% compared to median 98.0%; IQR: 96.0 to 99.0%) (Additional file 1: Table S4). The initial qSOFA score was higher among fatal cases (median 1.5; IQR: 1.0 to 2.0) compared to non-fatal cases (median 1.0; IQR: 0.0 to 1.0). Half of fatal cases had an initial qSOFA score ≥ 2 compared to 16.7% of non-fatal cases. The median ALT and AST were 26.0 U/L (IQR: 22.25 to 48.50) and 80.0 U/L (IQR: 34.75 to 215.75), respectively, which were higher (ALT p = 0.031; AST p = 0.001) among fatal cases than non-fatal cases (Additional file 1: Table S5). The majority (n = 14; 87.5%) of fatal cases had blood cultures without growth. All fatal cases had a negative malaria RDT (and one positive smear) compared to 32.4% of non-fatal cases had a positive malaria smear and 34.3% of non-fatal cases that had a positive malaria RDT (Table 2). There were 4 fatal cases (3 post-discharge) that had HIV and a positive urine LAM, highly suggestive of a diagnosis of tuberculosis. All LAM-positive cases received anti-tuberculosis treatment and one was treated for concomitant cryptococcal meningitis.

**Fig. 2:**
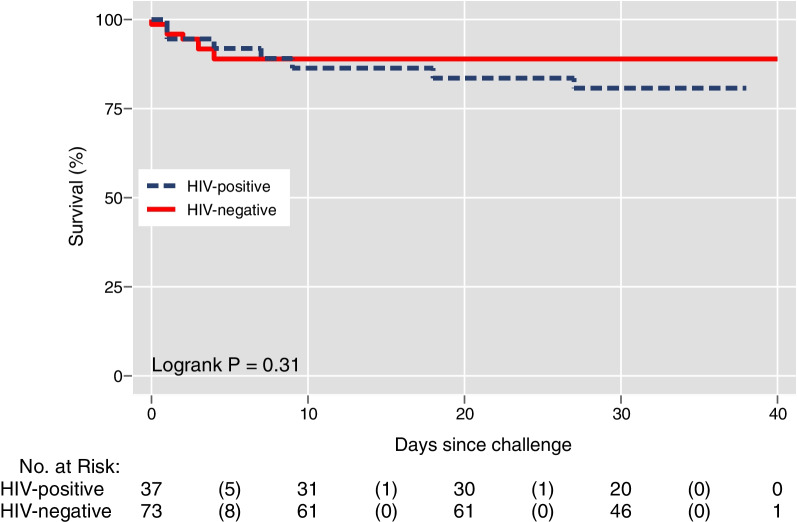
Kaplan–Meier plot of survival over time stratified by HIV status

**Fig. 3:**
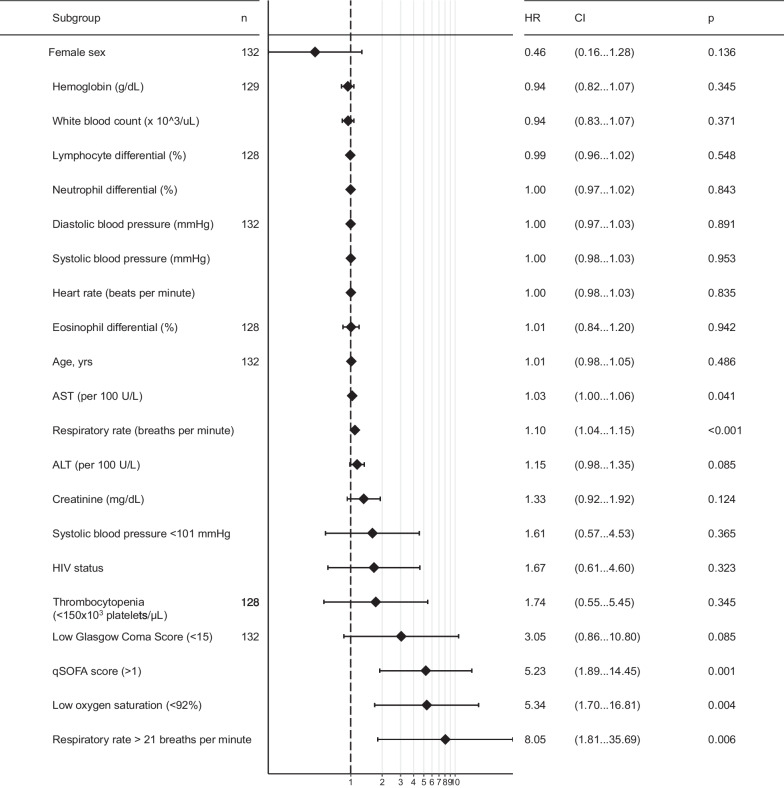
Forest plot of univariate unadjusted hazard ratio estimates of demographic characteristics, physiologic parameters, and clinical laboratory results for risk of death

Using Cox proportional hazards regression, risk of death was not found to be increased by age, sex, or HIV diagnosis (Fig. 2). HIV was not associated with risk of death (HR: 1.70; 95% CI 0.62 to 4.69; logrank p = 0.32). High respiratory rate (≥ 22 breaths per minute) (hazard ratio [HR] 8.05; 95% CI 1.81 to 35.69) and low (i.e., < 92%) oxygen saturation (HR 4.33; 95% CI 1.38 to 13.61) were identified to be associated with increased risk of death, but not heart rate (Fig. 3). Low (< 15) Glasgow Coma Score was associated with an increased risk of death (HR 3.05) but lacked statistical significance (95% CI 0.86 to 10.80). A high qSOFA (≥ 2 points) was associated with the largest increased risk of parameters evaluated (HR: 5.23; 95% CI 1.89 to 14.45). Clinical laboratory parameters were not associated with increased risk of death except for AST (HR: 1.03 per 100 U/L; 95% CI 1.00 to 1.06) and significance was not observed with ALT (HR: 1.15 per 100 U/L; 95% CI 0.98 to 1.35). The qSOFA score ≥ 2 and respiratory rate (continuous) parameters were selected based on a p cutoff < 0.002 for adjusted analyses. An elevated qSOFA remained associated (adjusted hazard ration [aHR]: 4.67; 95% CI 1.67 to 13.09) with an increased risk of death after adjustment for cohort site, age, and sex. The accuracy of qSOFA for predicting 28-day death was 0.68 (95% CI 0.56, 0.81) for the unadjusted model and 0.74 (95% CI 0.62 to 0.86) for the adjusted model. Respiratory rate also remained associated with increased risk (aHR: 1.09, 95%: 1.04 to 1.15) of death after adjustment. The accuracy of respiratory rate for predicting 28-day death was 0.77 (95% CI 0.66, 0.87) and 0.79 (95% CI 0.69 to 0.89) for the unadjusted and adjusted models, respectively.

## Discussion

In this prospective multi-site AFI cohort in rural Uganda, death occurred mostly among young adults with a low prevalence of baseline comorbid illness. Malaria and tuberculosis were common causes of febrile illness, but malaria was not a common cause of death. Among participants that died, clinical and laboratory features suggestive of systemic infections were observed including respiratory distress, an elevated qSOFA score, and elevated liver transaminases; a microbiologic diagnosis was not attained for most cases. Among deaths, over 40% occurred after hospital discharge and tuberculosis was the most common laboratory diagnosis. Our interim cohort results provide updated descriptive clinical epidemiology of febrile illness in the region and emphasize the need for more in-depth assessment of causes of death due to non-malarial causes of fever among hospitalized adults in Uganda. This has been urgently underscored by the subsequent and ongoing Ebola virus outbreak in Mubende, Uganda declared in September 2022.

While HIV infection remains common, the majority of febrile hospitalized adult participants did not have HIV. Our findings are consistent with recent studies suggesting that a lower proportion of febrile patients is due to HIV during the universal antiretroviral therapy era. The prevalence of HIV in this cohort was similar to that of hospitalized patients with febrile illness in the East African region in Tanzania ranging from 32% [25] to 39% in 2007 to 2008 [26]. However, HIV prevalence was 85% among septic patients in Uganda in a 2008 to 2009 cohort [27]. A more recent sepsis cohort from 2017 to 2019 had less but still a majority (55%) of participants had HIV [28]. Similarly, the majority (67%) of participants had HIV in a sepsis cohort in Malawi from 2018 to 2019 [29]. HIV prevalence in our cohort may be lower than previous cohorts in Uganda due to different level of severity in rural settings. However, we did not detect a difference in severity between those with and without HIV. Therefore, this trend in lower prevalence of HIV could represent decreased trends after the initiation of a ‘treat all’ approach. While larger studies and weighting with population-level demographics would be needed for incidence conclusions, this suggests that opportunistic infections may no longer represent the most common causes of febrile illness requiring hospitalization in Uganda. Malaria was a common infection but not a common cause of death, potentially due to frequent empirical treatment or availability of diagnostics. However, larger studies would be needed to confirm these findings. Importantly, however, tuberculosis was identified as a common cause of illness in this cohort and remains a major cause of severe infectious illness regardless of HIV status.

Overall, 16 new diagnoses of HIV were identified in this prospective cohort with universal HIV testing, emphasizing the importance of continuing to test all hospitalized febrile patients for HIV. These findings emphasize that HIV testing is high yield for hospitalized adult patients with febrile illness and unknown HIV serostatus in Uganda. Additionally, this provides an opportunity to leverage existing resources for linkage to HIV care per national standards. The deaths that occurred among people with HIV after discharge additionally highlights the need for continuity of care resources particularly among severely ill patients with HIV.

Respiratory dysfunction noted by respiratory rate and oxygen saturation was identified as a major risk factor for death in this cohort. While respiratory rate is a known prognosticators as part of the qSOFA score [30], lack of access to respiratory support at these clinical sites may have been a factor leading to fatal outcomes. Similar to other referral hospitals in SSA, access to invasive and non-invasive respiratory support, including highly skilled nursing, high flow oxygen, and ventilatory equipment was not readily available at these hospitals. Improved access to respiratory support may decrease hospitalized deaths due to infection.

While common, the majority of febrile illness was non-malarial and deaths were primarily non-malarial. The low rates of death among those with malaria may be due to longstanding immunity, early empirical treatment, and limited clinical effects of low burden infections. However, larger studies would be needed to confirm these findings.

Our study has notable limitations. First, participants were lost to follow-up (10.6% of the cohort) and causes of death after discharge were unknown. Standardized verbal autopsies were not performed. Participant attrition was in part due to hesitation to return to medical centres during the COVID-19 pandemic. Causes of death were not ascertainable with telephone follow-up. Second, the sample size is limited and associations with mortality that exist may not have been observed in our data. For example, there was no survival difference observed with HIV, but we present here our interim analysis observations and smaller effect sizes may be observed in a larger cohort. Sensitivity analyses restricted to site were not performed given limited number of fatal outcomes (n = 5) at the Arua site. Third, diagnostic test results reported here did not have extensive diagnostic testing with serology or multiplex PCR. However, testing was protocolized and in line with recommendations for rapid diagnostic testing and diagnostic assays currently available in Uganda. More expansive diagnostic testing is ongoing to understand the aetiologies of the unknown causes of febrile illness and death including emerging or neglected causes of illness including viral and rickettsial infections. Interim results are presented here to provide urgently needed updated descriptive epidemiologic information in a region with diverse causes of acute febrile illness.

Taken together, these findings highlight the need to strengthen clinical and microbiology laboratory testing capacity to identify the aetiologies of acute febrile illness, particularly in settings where the epidemiology of febrile illness is shifting. These results are mostly from the pre-COVID era and aetiologies of fever may have directly or indirectly changed. Evaluation of optimization of follow-up, treatments, or diagnoses are urgent areas of study to improve outcomes among hospitalized adults in SSA.

## Conclusions

The majority of deaths occurred among those less than 50 years of age and due to non-malarial febrile illness. Rickettsial and viral aetiologies of severe febrile illness may represent an undetected and pervasive burden of disease [31, 32]. Urgent surveillance efforts and field-ready clinical diagnostics are needed to identify treatable causes of mortality.

## Supplementary Information

**Additional file 1: Figure S1. **Enrollment and follow-up flow diagram. **Figure S2**. Map of Uganda with districts represented in cohorts and district population sizes. Triangle shape pins: original districts of participants that were enrolled at Arua Regional Referral Hospital. Black circle pins: districts of participants that were enrolled at Mubende Regional Referral Hospital. Map created using ArcGIS Online [GIS software]. Sources: Esri, HERE, Garmin, FAO, NOAA, USGS. **Table S1. **Symptoms at enrollment. **Table S2.** Clinical laboratory parameters. **Table S3. **Comparison of baseline characteristics between those with and without HIV. **Table S4. **Characteristics of fatal cases. **Table S5. **Initial laboratory values and microbiologic results stratified by fatal outcome.

## Data Availability

The data that support the study findings may be available upon reasonable request to the corresponding author.
