# Supplementary material for: Aetiology of hospitalized fever and risk of death at Arua and Mubende tertiary care hospitals in Uganda from August 2019 to August 2020
Source: BMC Infect Dis. 2022 Nov 21;22:869. doi: 10.1186/s12879-022-07877-3 (PMC9680122; doi:10.1186/s12879-022-07877-3)
Supplement: Supplementary file 1 — Additional file 1: Figure S1. Enrollment and follow-up flow diagram. Figure S2. Map of Uganda with districts represented in cohorts and district population sizes. Triangle shape pins: original districts of participants that were enrolled at Arua Regional Referral Hospital. Black circle pins: districts of participants that were enrolled at Mubende Regional Referral Hospital. Map created using ArcGIS Online [GIS software]. Sources: Esri, HERE, Garmin, FAO, NOAA, USGS. Table S1. Symptoms at enrollment. Table S2. Clinical laboratory parameters. Table S3. Comparison of baseline characteristics between those with and without HIV. Table S4. Characteristics of fatal cases. Table S5. Initial laboratory values and microbiologic results stratified by fatal outcome. [file 12879_2022_7877_MOESM1_ESM.docx]

**Figure S1.** Enrollment and follow-up flow diagram.


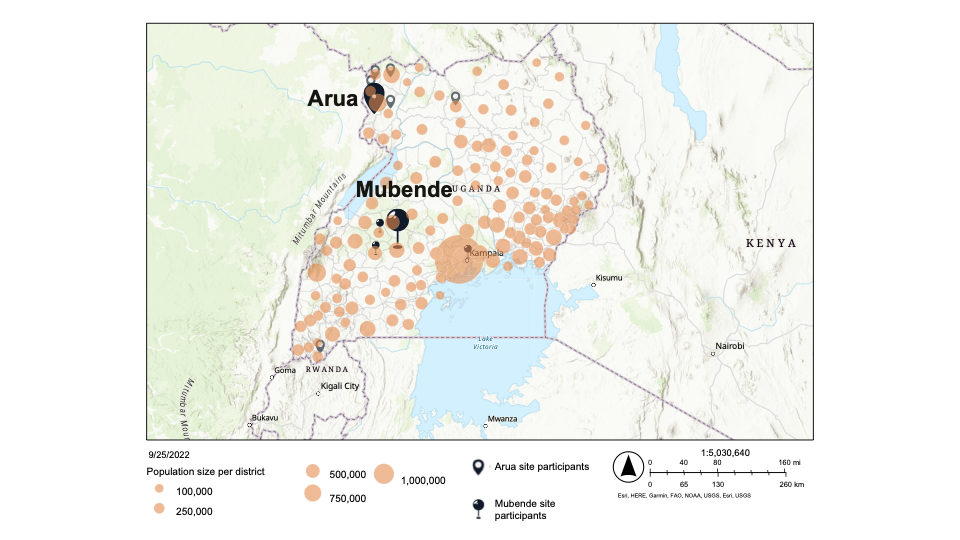


| **Figure S2**. Map of Uganda with districts represented in cohorts and district population sizes. Triangle shape pins: original districts of participants that were enrolled at Arua Regional Referral Hospital. Black circle pins: districts of participants that were enrolled at Mubende Regional Referral Hospital. Map created using ArcGIS Online [GIS software]. Sources: Esri, HERE, Garmin, FAO, NOAA, USGS. | | | |
| --- | --- | --- | --- |
| **Table S1.** Symptoms at enrollment. | | | |
|  | HIV-negative (n=87) | HIV-positive (n=45) |  |
| **Symptom — no. (%)** |  |  |  |
| Headache | 67 (77.0%) | 33 (73.3%) |  |
| Abdominal pain | 52 (59.8%) | 21 (46.7%) |  |
| Anorexia | 52 (59.8%) | 27 (60.0%) |  |
| Nausea/vomiting | 48 (55.2%) | 29 (64.4%) |  |
| Arthralgias | 41 (47.1%) | 15 (33.3%) |  |
| Fatigue | 39 (44.8%) | 18 (40.0%) |  |
| Rigors | 36 (41.4%) | 17 (37.8%) |  |
| Other | 34 (39.1%) | 13 (28.9%) |  |
| Myalgias | 33 (37.9%) | 11 (24.4%) |  |
| Cough | 26 (29.9%) | 20 (44.4%) |  |
| Diarrhea (> 3 stools/24hrs) | 20 (23.0%) | 19 (42.2%) |  |
| Dysuria | 13 (14.9%) | 5 (11.1%) |  |
| Bleeding from any site | 8 (9.2%) | 5 (11.1%) |  |
| Dyspnea | 7 (8.0%) | 5 (11.1%) |  |
| Altered mental status | 6 (6.9%) | 7 (15.6%) |  |
| Sore throat | 6 (6.9%) | 2 (4.4%) |  |
| Rash | 1 (1.1%) | 3 (6.7%) |  |
| None | 0 (0.0%) | 1 (2.2%) |  |

| **Table S2.** Clinical laboratory parameters. | | | | | |
| --- | --- | --- | --- | --- | --- |
| Parameter | Total (n=132) | HIV-negative (n=87) | HIV-positive (n=45) | p value |  |
| **Hematology — median (IQR)** |  |  |  |  |  |
| Hemoglobin (g/dL) | 12.6 (9.7, 14.0)* | 12.5 (8.9, 14.0) | 12.8(10.6, 13.8) | 0.351 |  |
| Platelet count (cells x 10^3^/uL) | 129.0 (73.0, 184.0)** | 116.0 (59.0, 180.5) | 147.0 (98.0, 187.0) | 0.140 |  |
| White blood count (cells x 10^3^/uL) | 4.8 (3.2, 8.1)* | 5.2 (3.5, 8.4) | 4.4 (2.6, 7.5) | 0.322 |  |
| **White blood count differential — %, median (IQR)** |  |  |  |  |  |
| Neutrophils | 57.3 (40.7, 69.3)** | 59.3 (41.5, 69.0) | 57.0 (39.6, 72.1) | 0.925 |  |
| Lymphocytes | 29. 0 (19.0, 43.5)** | 28.1 (19.1, 44.6) | 32.2 (18.1, 40.9) | 0.621 |  |
| Eosinophils | 1.0(0.8, 1.7)** | 1.0 (0.8, 1.7) | 1.1 (0.9, 1.6) | 0.374 |  |
| **CD4 count —cells/mL, median (IQR)** | 198.0 (72.0, 347.0) | NA | 198.0 (72.000, 347.000) |  |  |
| **Serum chemistry parameters —mg/dL, median (IQR)** |  |  |  |  |  |
| **Creatinine** | 0.8 (0.7, 1.1) | 0.800 (0.7, 1.1) | 0.900 (0.7, 1.1) | 0.206 |  |
| **ALT** | 21.0 (14.0, 34.5) | 22.0 (14.0, 37.5) | 21.0 (14.0, 30.0) | 0.582 |  |
| **AST** | 32.0 (24.0, 78.8) | 32.0 (23.0, 88.5) | 32.0 (28.0, 59.0) | 0.943 |  |

ALT: alanine transaminase; AST: aspartate transaminase

*Missing from 3 participants due to technical issues. **Missing from 4 participants due to technical issues.

| **Table S3.** Comparison of baseline characteristics between those with and without HIV. | | | |
| --- | --- | --- | --- |
| Parameter | HIV-negative (n=87) | HIV-positive (n=45) | p value |
| **Female sex — no. (%)** | 50 (57.5%) | 27 (60.0%) | 0.780 |
| **Age— years, median (IQR)** | 30.0 (22.0, 44.5) | 38.0 (30.0, 48.0) | **0.005** |
| **Past medical history — no. (%)** |  |  |  |
| COPD or asthma | 1 (1.1%) | 0 (0.0%) | 0.470 |
| Diabetes mellitus | 3 (3.4%) | 2 (4.4%) | 0.776 |
| Sickle cell disease/thalassemia | 3 (3.4%) | 0 (0.0%) | 0.208 |
| **Duration of symptoms — days, median (IQR)** | 4.0 (3.0, 7.0) | 5.0 (3.0, 7.0) | 0.051 |
| **Physiologic parameters — median (IQR)** |  |  |  |
| Heart rate (beats per minute) | 107.0 (93.0, 119.5) | 111.0 (93.0, 118.0) | 0.925 |
| Temperature (degrees Centigrade) | 38.3 (38.0, 38.8) | 38.1 (38.0, 38.7) | 0.170 |
| Systolic blood pressure (mmHg) | 110.0 (100.0, 119.0) | 114.0 (98.0, 127.0) | 0.236 |
| Diastolic blood pressure (mmHg) | 68.0 (59.0, 75.0) | 69.0 (63.0, 80.0) | 0.127 |
| Respiratory rate (breaths per minute) | 22.0 (18.0, 25.5) | 20.0 (18.0, 24.0) | 0.712 |
| Oxygen saturation (%) | 98.0 (96.0, 98.0) | 98.0 (96.0, 99.0) | 0.524 |
| Glasgow coma scale | 15.0 (15.0, 15.0) | 15.0 (15.0, 15.0) | 0.810 |
| **qSOFA score ≥2 — no. (%)** | 19 (21.8%) | 8 (17.8%) | 0.583 |

|  |  |  |  |  |
| --- | --- | --- | --- | --- |
| **Table S4.** Characteristics of fatal cases. | | | |  |
| Characteristic | | Survived (n=102) | Died (n=16) |  |
| **Female sex — no. (%)** | | 60 (58.8%) | 7 (43.8%) |  |
| **Age — years, median (IQR)** | | 32.0 (24.0, 46.8) | 38.0 (29.5, 41.3) |  |
| **Past medical history — no. (%)** | |  |  |  |
| COPD or asthma | | 1 (1.0%) | 0 (0.0%) |  |
| Diabetes mellitus | | 5 (4.9%) | 0 (0.0%) |  |
| Sickle cell disease/thalassemia | | 3 (2.9%) | 0 (0.0%) |  |
| **HIV-positive — no. (%)** | | 31 (30.4%) | 7 (43.8%) |  |
| **Duration of symptoms — days, median (IQR)** | | 4.0 (3.0, 7.0) | 6.0 (3.0, 7.0) |  |
| **Physiologic parameters — median (IQR)** | | 105.0 (92.0, 116.8) | 115.5 (95.3, 126.3) |  |
| Heart rate (beats per minute) | |  |  |  |
| Temperature (degrees Centigrade) | | 38.1 (38.0, 38.7) | 38.6 (37.7, 39.3) |  |
| Systolic blood pressure (mmHg) | | 111.5 (100.0, 120.8) | 112.0 (96.8, 122.8) |  |
| Diastolic blood pressure (mmHg) | | 68.0 (60.0, 76.8) | 69.000 (46.5, 84.0) |  |
| Respiratory rate (breaths per minute) | | 20.0 (18.0, 24.0) | 24.5 (22.0, 31.0) |  |
| Oxygen saturation (%) | | 98.0 (96.0, 99.0) | 95.5 (92.3, 98.0) |  |
| Glasgow coma scale | | 15.0 (15.0, 15.0) | 15.0 (15.0, 15.0) |  |
| **qSOFA score — median (IQR)** | | 1.0 (0.000, 1.0) | 1.5 (1.0, 2.0) |  |
| **qSOFA score ≥2 — no. (%)** | | 17 (16.7%) | 8 (50.0%) |  |
| **Symptom — no. (%)** | |  |  |  |
| Abdominal pain | | 59 (57.8%) | 6 (37.5%) |  |
| Altered mental status | | 7 (6.9%) | 5 (31.2%) |  |
| Anorexia | | 62 (60.8%) | 7 (43.8%) |  |
| Arthralgias | | 42 (41.2%) | 6 (37.5%) |  |
| Bleeding from any site | | 8 (7.8%) | 2 (12.5%) |  |
| Dyspnea | | 8 (7.8%) | 3 (18.8%) |  |
| Cough | | 29 (28.4%) | 9 (56.2%) |  |
| Diarrhea (> 3 stools/24hrs) | | 30 (29.4%) | 4 (25.0%) |  |
| Dysuria | | 15 (14.7%) | 2 (12.5%) |  |
| Fatigue | | 44 (43.1%) | 5 (31.2%) |  |
| Headache | | 77 (75.5%) | 11 (68.8%) |  |
| Myalgias | | 33 (32.4%) | 3 (18.8%) |  |
| Nausea/vomiting | | 60 (58.8%) | 8 (50.0%) |  |
| Rash | | 3 (2.9%) | 0 (0.0%) |  |
| Rigors | | 44 (43.1%) | 2 (12.5%) |  |
| Sore throat | | 7 (6.9%) | 1 (6.2%) |  |
| Other | | 34 (33.3%) | 7 (43.8%) |  |
| None | | 1 (1.0%) | 0 (0.0%) |  |

| **Table S5.** Initial laboratory values and microbiologic results stratified by fatal outcome.* | | |
| --- | --- | --- |
| Parameter | Survived (n=102) | Died (n=16) |
| **Hematology — median (IQR)** |  |  |
| Hemoglobin (g/dL) | 12.9 (10.8, 14.5)** | 11.0 (8.8, 12.8) |
| Platelet count (cells x 10^3^/uL) | 134.0 (77.0, 184.5)* | 107.0 (71.8, 155.0) |
| White blood count (cells x 10^3^/uL) | 5.1 (3.3, 7.8)** | 4.4 (3.0, 7.7) |
| **White blood count differential — %, median (IQR)** |  |  |
| Neutrophils | 58.7 (42.0, 71.2)** | 59.5 (38.9, 64.6) |
| Lymphocytes | 31.0 (18.7, 43.0)** | 27.1 (21.0, 40.5) |
| Eosinophils | 1.1 (0.9, 1.7) ** | 0.9 (0.6, 1.3) |
| **CD4 count —cells/mL, median (IQR)** | 219.0 (114.0, 402.5) | 69.0 (40.0, 119.0) |
| Total no. | 31 | 7 |
| **Serum chemistry parameters —mg/dL, median (IQR)** |  |  |
| Creatinine | 0.8 (0.7, 1.1) | 1.1 (0.8, 1.6) |
| ALT | 20.5 (14.0, 32.8) | 26.0 (22.3, 48.5) |
| AST | 30.5 (22.0, 61.3) | 80.0 (34.8, 215.8) |

ALT: alanine transaminase; AST: aspartate transaminase

*Excludes those lost to follow-up.

**White blood count and hemoglobin not available from 3 participants due to technical issues (e.g., clotting). Cell differential not available from 4 participants.
